# Influence of Estradiol-17beta on Progesterone and Estrogen Receptor mRNA Expression in Porcine Follicular Granulosa Cells during Short-Term,* In Vitro* Real-Time Cell Proliferation

**DOI:** 10.1155/2016/8431018

**Published:** 2016-12-26

**Authors:** Sylwia Ciesiółka, Joanna Budna, Karol Jopek, Artur Bryja, Wiesława Kranc, Adrian Chachuła, Sylwia Borys, Marta Dyszkiewicz Konwińska, Agnieszka Ziółkowska, Paweł Antosik, Dorota Bukowska, Klaus P. Brüssow, Małgorzata Bruska, Michał Nowicki, Maciej Zabel, Bartosz Kempisty

**Affiliations:** ^1^Department of Histology and Embryology, Poznań University of Medical Sciences, 6 Święcickiego St., 60-781 Poznań, Poland; ^2^Department of Anatomy, Poznań University of Medical Sciences, 6 Święcickiego St., 60-781 Poznań, Poland; ^3^Department of Biomaterials and Experimental Dentistry, Poznań University of Medical Sciences, 70 Bukowska St., 60-812 Poznań, Poland; ^4^Department of Anatomy and Histology, Faculty of Medicine and Health Sciences, University of Zielona Gora, ul. Zyty 28, 65-046 Zielona Gora, Poland; ^5^Institute of Veterinary and Animal Science, Poznań University of Life Sciences, 52 Wojska Polskiego St., 60-628 Poznań, Poland; ^6^Department of Histology and Embryology, Wroclaw Medical University, 6a Chalubinskiego St., 50-368 Wroclaw, Poland

## Abstract

Progesterone (P4) and estradiol (E2) play a significant role in mammalian reproduction. Our study demonstrated that separated porcine cumulus cells (CCs) and/or granulosa cells (GCs) might proliferate* in vitro* during short-term, real-time primary culture. The GCs were analyzed according to gene expression of the progesterone receptor (nuclear form) (*pgr*), progesterone receptor membrane component 1 (*pgrmc1*), and estrogen-related receptor beta 3 (*esrrb3*) in relation to two housekeeping genes:* actb* and* pbgd*. GCs were cultivated in medium with the E2. Both* pgr*/*actb* and* pgr*/*pbgd* revealed higher expression between 24 and 168 h of IVC of prolonged E2 treatment and at 48 h of IVC after acute E2 administration. The* pgrmc1*/*actb* and* pgrmc1*/*pbgd* displayed increased expression after prolonged E2 treatment between 24 and 120 h of IVC. The highest level of* esrrb3*/*actb* at 120 and 144 h, as well as* esrrb3*/*pbgd* at 120 h, in untreated controls as compared to the hormone-stimulated group, was observed. We suggest that E2 significantly influences the upregulation of* pgr*,* pgrmc1*, and* esrrb3* expression in porcine GCs during real-time cell proliferation. Since* esrrb3* expression is stimulated by E2 in both an acute and prolonged manner, estradiol may be recognized as a potential estrogen receptor agonist in GCs.

## 1. Introduction

Folliculogenesis is a compound process, involving several biochemical and morphological changes, ultimately leading to antral follicle formation. The antral follicle is comprised of theca and granulosa cell layers that surround the growing follicle. It acts to support the growth and development of oocyte as well as cumulus cell layer differentiation [1]. It was demonstrated by Knight and Glister that growing oocytes, granulosa cells (GCs), a basement membrane, and theca cells (TCs), as well as blood capillaries, are involved in the process of follicle-oocyte crosstalk that includes small metabolite transition [2]. The main proteins expressed and transferred between these structures include factors belonging to the TGFB superfamily such as BMP-2,-5,-6, BMP-4, BMP-7, BMP-15, GDF-9, inhibin, activin, and AMH. Moreover, it was demonstrated that an LH surge leads to increased expression of progesterone receptor (PGR). This mechanism activates the transfer of a disintegrin-like and metallopeptidase (reprolysin type) with thrombospondin type 1 (ADAMTS1) into cumulus cells (CCs), granulosa, and theca cell layers. Increased expression of ADAMTS1 activates the transfer and expression of EGFp in cumulus cells [3].

We recently showed that porcine cumulus-granulosa cells isolated from antral follicles could be kept in primary culture in a long-term cultivation system. During this period, a logarithmic increase of real-time proliferation of GCs was observed. Furthermore, significant expression changes of connexin 43 (CX43) and cyclin-dependent kinase 4 (CDK4) mRNA were determined [4]. We also found translocation of CX43 and CDK4 proteins between the cell nucleus, membrane, and cytoplasm. These experiments proved that proliferation is highly correlated with the distribution of proteins between the nucleus and cytoplasm. Hence, it may be a key factor influencing the processes of nuclear-cytoplasmic shuttling, bidirectional transport of selected proteins, for example, those responsible for oocyte-cumulus dialog, and the control of cell cycle divisions.

Progesterone (P4) and estradiol-17beta (E2) are steroid hormones that regulate important phases of the reproductive cycle including oocyte growth and maturation and follicle development from the preantral to antral stage, as well as differentiation of follicular theca, granulosa, and cumulus cells. Both of these steroids act* via* specific nuclear receptors that form specific complexes that bind to promoter response elements and regulate target gene expression. Furthermore, membrane receptor components for example, PGRMC1 (progesterone receptor membrane component 1), which binds agonists on the cell membrane surface and then activates signal transduction pathways through the cytoplasm into the nucleus, also belong to this group of steroid receptors.

The role of P4 and E2 in regulation of the ovulatory cycle is widely recognized; however, it still remains unclear whether E2 may significantly influence progesterone receptors and/or estrogen receptor-related protein expression in porcine follicular granulosa cells. Therefore, the goal of this study was to compare the association between the expression of nuclear and membrane isoforms of progesterone receptor (PGR and PGRMC1) and ESRRB3 in relation to real-time proliferation of porcine follicular granulosa cells in a primary culture model.

## 2. Material and Methods

### 2.1. Animals

A total of 30 crossbred Landrace gilts with a median age of 120 days and a median weight of 98 kg were used in this study. All of the animals were kept under the same housing conditions. All experiments were approved by the local ethics committee.

### 2.2. Collection of Porcine Ovaries and* In Vitro* Cultivation of Porcine Granulosa Cells (GCs)

The ovaries and reproductive tracts were recovered immediately after slaughter and transported within 40 minutes to the laboratory in a solution of 0.9% NaCl (38°C). The ovaries of each animal were placed in 5% fetal bovine serum (FBS; Sigma-Aldrich Co., St. Louis, MO, USA) in PBS. Granulosa cells with cumulus-oocyte complexes (COCs) were collected by aspirating the follicular fluid from single large follicles (>5 mm) using a 20 G needle. COCs were removed from the cell suspension by filtration through a 40 *μ*m cell strainer and discarded. To obtain GCs follicular fluid was centrifuged at 200 ×g for 10 min at RT. Subsequently GCs were washed by centrifugation at 200 ×g for 10 min at RT with culture medium. The* in vitro* culture (IVC) medium consisted of Dulbecco's Modified Eagle's Medium (DMEM/F12, Sigma-Aldrich, USA), 2% fetal calf serum FCS (PAA, Austria), 10 mg/mL ascorbic acid (Sigma-Aldrich, USA), 0.05 *μ*M dexamethasone (Sigma-Aldrich, USA), 200 mM L-glutamine (Invitrogen, USA), 10 mg/mL gentamicin (Invitrogen, USA), 10,000 units/mL penicillin, and 10,000 *μ*g/mL streptomycin (Invitrogen, USA). We used 3 × 10^6^ cells per dish for culture and 5 × 10^4^ cells per one E-Plate in real-time cell analyzer (RTCA) system. To remove dead cells, plates were washed twice with culture medium after 24 h of IVC. Cells were cultivated for 168 h with 1.0 *μ*g/mL E2 once added at 48 h of IVC (acute treatment) and at 0 h of IVC (prolonged treatment). Cells were cultivated at 38.5°C under aerobic conditions (5% CO_2_). Once the adherent cells were more than 80% confluent, they were detached with 0.05% trypsin-EDTA (Invitrogen, USA) for 10 min and counted using a Z2 counter or cell viability analyzer (Vi-Cell XR 2.03; both Beckman Coulter, USA).

### 2.3. *In Vitro* GC Cultivation Using a Real-Time Cellular Analyzer (RTCA)

The recovered GCs were transferred into an E-Plate 16 of a real-time cell analyzer (RTCA, Roche-Applied Science, GmbH, Penzberg, Germany), which consisted of an RTCA analyzer, an RTCA SP station, and RTCA software. The GCs were then cultured in 200 *μ*L of standard porcine IVC medium that consisted of Dulbecco's Modified Eagle's Medium (DMEM/F12, Sigma-Aldrich, USA) as mentioned above. The GCs were cultured for 0–168 h at 38.5°C under 5% CO_2_ in air. Group A is control group without E2 and rest of groups were treated with E2 for 168 h. After each cultivation period, the cell index (CI) was used to evaluate the relative and quantitative changes in electrical cell impedance. The cell status was determined using the RTCA software.

### 2.4. Real-Time Quantitative Polymerase Chain Reaction (RT-qPCR) Analysis

Total RNA was isolated from GCs before (at 0 h) and after (24, 48, 72, 96, 120, 144, and 168 h) IVC using an RNeasy mini column from Qiagen GmbH (Hilden, Germany). The RNA samples were resuspended in 20 *μ*L of RNase-free water and stored in liquid nitrogen. RNA samples were then treated with DNase I and reverse-transcribed (RT) into cDNA. RQ-PCR was conducted in a LightCycler real-time PCR detection system (Roche Diagnostics GmbH, Mannheim, Germany) using SYBR® Green I as a detection dye, and target cDNA was quantified using the relative quantification method. The relative abundance of PGR, PGRMC1, and ESRRB3 transcripts in each sample was standardized to the internal standards ACTB and PBGD. For amplification, 2 *μ*L of cDNA solution was added to 18 *μ*L of QuantiTect® SYBR Green PCR (Master Mix Qiagen GmbH, Hilden, Germany) and primers (Table 1). One RNA sample of each preparation was processed without the RT-reaction to provide a negative control for subsequent PCR.

To quantify the specific genes expressed in GCs, expression levels of specific oocyte mRNAs were calculated related to* pbgd* and* actb*. To ensure the integrity of these results, the additional housekeeping gene 18S rRNA was used as an internal standard to demonstrate that* pbgd* and* actb* mRNAs were not differentially regulated in the groups of GCs. The gene for 18S rRNA expression has been identified as an appropriate housekeeping gene for use in quantitative PCR studies. Expression of* pbgd*,* actb*, and 18S rRNA mRNA was measured in cDNA samples from isolated GCs.

### 2.5. Statistical Analysis

A one-way ANOVA, followed by Tukey's* post hoc* test, was used to compare the results of the real-time quantification of the proliferation index. The experiments were carried out in at least two replicates. The results quantifying the cell proliferation index were obtained using an RTCA system. The differences were considered to be significant at ^*∗*^ *P* < 0.05, ^*∗∗*^ *P* < 0.01, and ^*∗∗∗*^ *P* < 0.001 for the RT-qPCR and RTCA analyses. They were evaluated by comparing the results obtained in five replicates of the same recovered granulosa cells. Statistical calculations were applied to the highest normalized proliferation index at each time point for each investigated group to compare the results. All statistical analyses were performed using the software program GraphPad Prism version 4.0 (GraphPad Software, San Diego, CA).

## 3. Results

**Figure 1:**
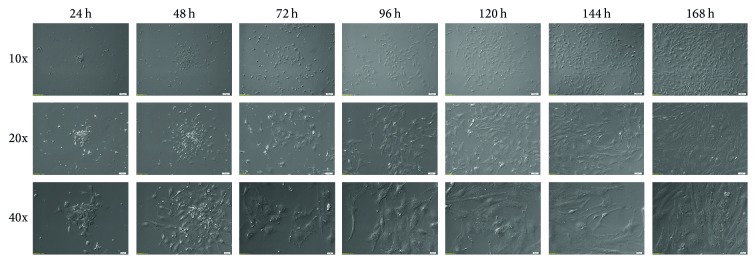
Representative picture of changes in cell morphology during short-term (168 h) primary cell culture. The cells isolated from porcine ovarian follicles were cultured for 168 h. Each 24 h cells growth and morphology changes were documented by Nomarski system with 10x, 20x, and 40x objective lenses (total magnifications: 6,3x, 12,6x, and 25,2x, resp.; *M*_total_ = *M*_objective_ × *M*_video  camera  adapter_).

The changes in cell morphology cultured* in vitro* for 168 h were analyzed by Nomarski system (Figure 1).

**Figure 2:**
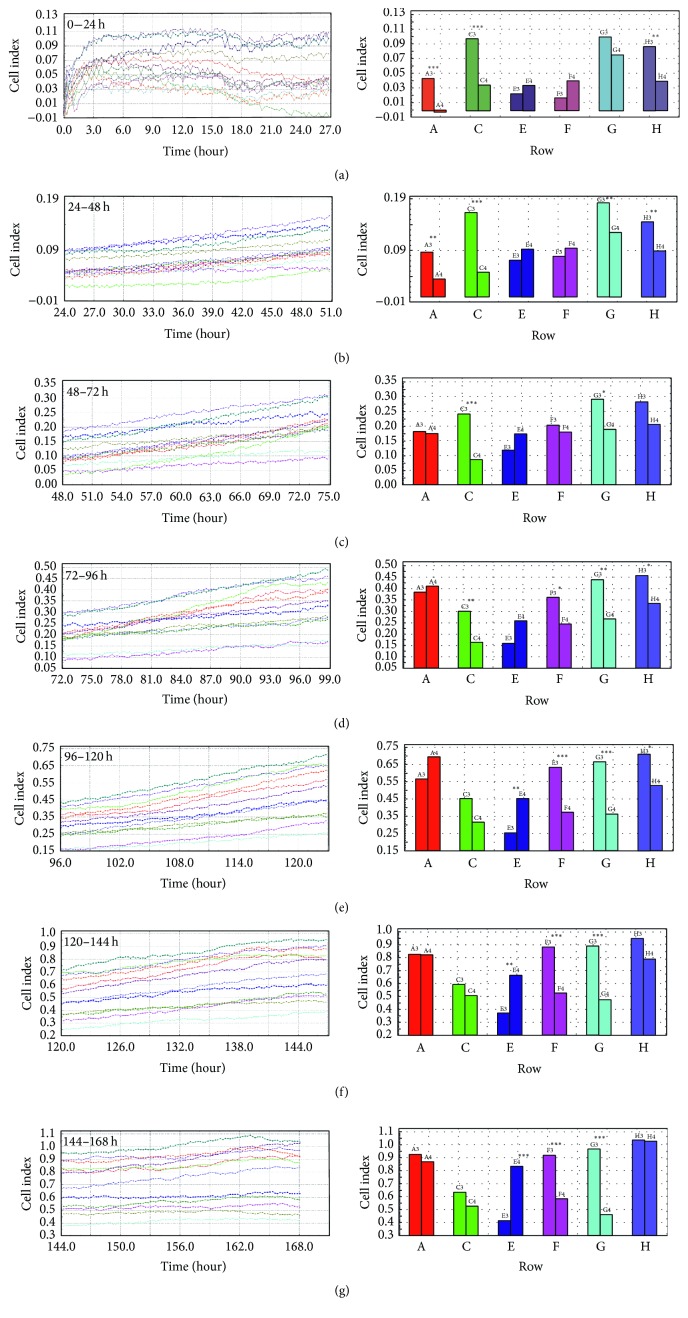
Cell proliferation index (CI) of porcine follicular granulosa cells cultivated for 168 h. Porcine follicular granulosa cells were recovered from pubertal gilts and treated with collagenase for 10 min at 38.5°C. The cells were immediately transferred into an E-Plate 48 of a real-time cell analyzer (RTCA, Roche-Applied Science, GmbH, Penzberg, Germany). The experiment consisted of six groups (2 replicates in each group) involving the cultivation of the same population of collected cells. Column A represents control group and rest of groups were treated with E2 for prolonged time. At each step, the cell proliferation index (CI) was assessed for real-time* in vitro* cultivation for the time periods 0–24 h (a), 24–48 h (b), 48–72 h (c), 72–96 h (d), 96–120 h (e), 120–144 h (f), and 144–168 h (g). CI is an unitless parameter calculated, based on impedance of electron flow caused by adherent cells, CI = (impedance at time point *n*  − impedance in the absence of cell)/nominal impedance value. The differences were considered to be significant at the level of ^*∗*^ *P* < 0.05, ^*∗∗*^ *P* < 0.01, and ^*∗∗∗*^ *P* < 0.001.

During short-term cell cultivation and prolonged E2 treatment, the porcine follicular granulosa cells were kept in the RTCA system for 168 h. The cell proliferation index (PI) was analyzed after each 24 h period between 0 and 168 h, with measurements of cell proliferation progressing every 15 minutes. The PI was analyzed between 0–24 h, 24–48 h, 48–72 h, 72–96 h, 96–120 h, 120–144 h, and 144–168 h, and the differences in cell proliferation progression were evaluated in six groups with at least two replicates for each. At first stage (0–24 h), when cells were at lag phase, we observed differences in PI between three of the six groups (*P* < 0.001 for two groups and *P* < 0.01) (Figure 2(a)). Analysis of the cell proliferation index after 24–48 h of cultivation showed proliferation differences in four of six groups at the level of significance *P* < 0.01 for three and *P* < 0.001 for one of the groups (Figure 2(b)). During the log phase of cellular proliferation, between 48 and 72 h, we found differences in PI in two of six groups (*P* < 0.001 and *P* < 0.05, resp.) (Figure 2(c)). After examination of PI between 72 and 96 h, 96 and 120 h, and 120 and 144 h, we observed differences in four of the six groups (between 72–96 h and 96–120 h) and in three of the six groups (between 120–144 h), respectively, with the levels of significance at *P* < 0.05 (Figures 2(d), 2(e), and 2(f)). At 144–168 h, the differences in three of six groups were found with levels of significance at *P* < 0.001 for each (Figure 2(g)).

**Figure 3:**
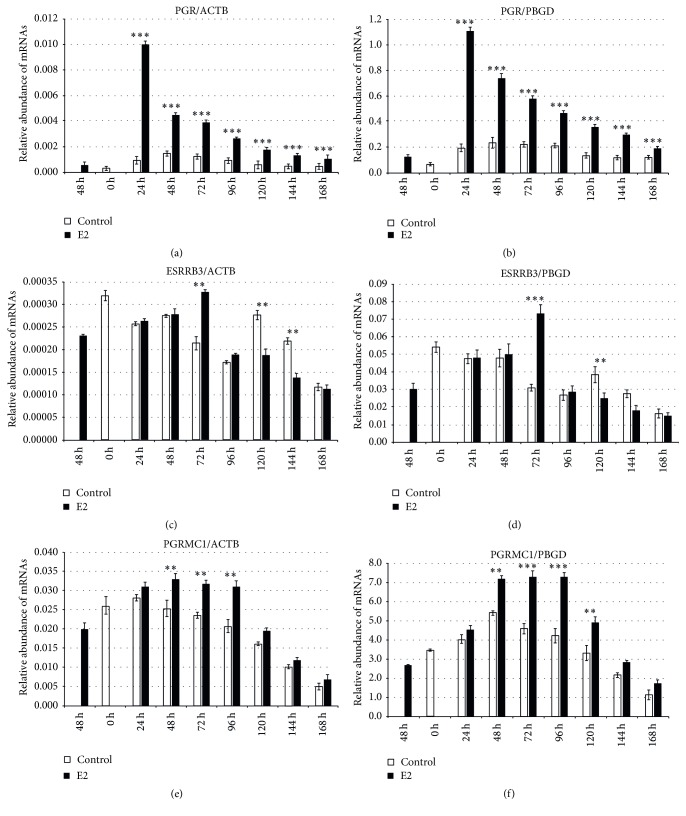
Relative abundance of* pgr*,* esrrb3*, and* pgrmc1* transcripts in porcine follicular granulosa cells analyzed during 168 h of IVC in relation to* actb* and* pbgd* housekeeping genes. Porcine follicular granulosa cells were isolated from pubertal gilts and immediately used to isolate RNA, which was reverse-transcribed into cDNA. The relative abundance of* pgr* (a and b),* esrrb3* (c and d), and* pgrmc1* (e and f) mRNAs was evaluated by RT-qPCR analysis before and after each 24 h of IVC in relation to two housekeeping genes:* actb* and* pbgd*, respectively. The results are presented as the mean ± SEM with the level of significance shown as ^*∗∗*^ *P* < 0.01 and ^*∗∗∗*^ *P* < 0.001.

Using RT-qPCR analysis, we assessed the expression of steroid-related receptors such as PGR, ESRRB3, and PGRMC1 after acute and prolonged E2 treatment during GCs short-term, real-time proliferation* in vitro*. Transcript expression was compared to two housekeeping genes:* actb* and* pbgd*. We did not find increased expression of* pgr* after acute E2 treatment (at 48 h of IVC) (*P* < 0.001), but after prolonged E2 treatment between 24 and 168 h of IVC expression of* pgr* has been increased (*P* < 0.001) (Figure 3(a)). However, the highest level of* pgr* was observed at 24 h of IVC in the E2 treated group (*P* < 0.001). A similar effect of E2 treatment on* pgr* expression was found when compared to* actb* and* pbgd* expression (Figures 3(a) and 3(b)). Analysis of* esrrb3*/*actb* expression revealed a higher level of mRNA after acute and 72 h of prolonged E2 treatment as compared to the control (*P* < 0.01) (Figure 3(c)). Similarly,* esrrb3*/*pbgd* levels were increased after acute E2 treatment and after 72 h of prolonged E2 administration as compared to the control (*P* < 0.001) (Figure 3(d)). The expression of* esrrb3*/*actb* and* esrrb3*/*pbgd* at 120 and 144 h was increased in the control group as compared to the hormone treated group (*P* < 0.01 for both, resp.). Similarly, higher* esrrb3* expression, according to both* actb* and* pbgd*, was observed at 0 h in the control group as compared to the E2 treated group (*P* < 0.001) (Figures 3(c) and 3(d)). We showed increased expression of* pgrmc1*/*actb* and* pgrmc1/pbgd* after acute E2 treatment as compared to the control (*P* < 0.001) (Figures 3(e) and 3(f)). At 0 h of IVC, an increased level of both* pgrmc1*/*actb* and* pgrmc1*/*pbgd* in the control as compared to E2 group was observed. The highest level of* pgrmc1*/*actb* in the E2 treated group was seen after 48, 72, and 96 h (*P* < 0.01 for all time periods) (Figure 3(e)). The* pgrmc1*/*pbgd* levels were more highly expressed at 48, 72, 96, and 120 h in the E2 treated group as compared to the control (*P* < 0.01, *P* < 0.001, *P* < 0.001, and *P* < 0.01, resp.) (Figure 3(f)).

## 4. Discussion

In mammals, proper COC maturation is accompanied by cumulus cell (CC) expansion. It is suggested that this unique event is required so that oocytes reach a full maturation stage (MII), furthering gamete interaction and fusion during monospermic fertilization. The CC expansion is orchestrated by substantial biochemical and morphological modifications of single oocytes and surrounding somatic cells. However, until now, it is not understood if CC expansion, either* in vivo* or* in vitro*, is accompanied by a substantial increase in cell proliferation. Our recent study proved that porcine CCs, separated from oocytes as well as cumulus-enclosed oocytes, may proliferate* in vitro* during short-term, real-time cultivation. Moreover, the significant increase of the proliferation index (PI) of granulosa cells (GCs) collected from porcine large ovarian follicles was also observed [4]. It is well known that the process of CC expansion in a cumulus-enclosed oocyte model is associated with the activation of CC-oocyte cross talk* via* gap junction connections (GJC). Indeed, it was also presented in our previous study, which demonstrated a substantial increase in connexin expression in porcine-separated GCs during real-time, short-term primary culture [5]. These experiments aided to confirm our recent hypothesis stating the activity of GJC in CC-enclosed oocytes, single CCs, and separated GCs. This observation brings a new insight into the metabolic activity of cells that surround oocytes such as CCs and/or GCs, which form the architecture of porcine ovarian follicles.

Steroid hormones play a significant role during mammalian COC maturation, single oocyte growth and development, and CC differentiation. Moreover, it is now well known that P4 and E2 substantially regulate the proper course of folliculogenesis and oogenesis. However, until now, it has not been clear if the P4 and/or E2 influences CC/GCs proliferation or differentiation* in vivo* or* in vitro*. Furthermore, the acute or prolonged effect of E2 administration on GC proliferation and steroid receptor expression requires further exploration.

In the current study, we investigated the prolonged effect of E2 administration on real-time GC proliferation during short-term,* in vitro* culture. We found a significant increase in the PI shortly after 48–72 h of IVC, which was maintained through the following periods of IVC. Several studies have shown that steroid hormones may decrease proliferative activity of cells* in vivo* and* in vitro* [6]. However, no data exists indicating the influence of E2 on porcine GC in* in vitro,* real-time proliferation. Results from the current study suggest that E2 treatment does not affect cell proliferation. Based on our previous observations, we postulate that the PI displays a similar pattern to the untreated group of GCs during IVC. It is also suggested that the increased PI between 48 and 72 h during GCs real-time culturing may be a result of granulosa cell differentiation into luteal cells, which takes place both* in vivo* and* in vitro.* This is accompanied by marked activity of gonadotropins, especially luteinizing hormone (LH) and follicle-stimulating hormone (FSH) and their receptors, LHR and FSHR. Our recent experiments (unpublished data) indicate the stimulatory effect of E2 after acute treatment at 96 h of porcine GC IVC. However, an increased level of* esrrb3* mRNA after 72 h of IVC was presented in the current study. The higher level of the* esrrb3* transcript may be a result of ligand availability in the extracellular matrix and/or increased activity of signal transduction pathways from the GC membrane into nuclear estrogen receptors.

It is well understood that progesterone plays a substantial role during mammalian ovarian folliculogenesis. A recent study by Durlej et al. was based on an immunohistochemical assessment of the distribution of two progesterone receptor isoforms, PGRA and PGRB, within porcine ovaries at various stages of development; in small and large antral follicles and early, midluteal, and regressing corpora lutea [7]. Both PGRs were present in GCs, TC, corpus luteum, and the epithelium surface. Moreover, in the antral follicles, the existence of PGRA was detected in theca interna cells, whereas, in large preovulatory follicles, PGRA was distributed in both granulosa and theca cell layers. Contrary to PGRA, PGRB was localized in GCs of antral follicles and was not detected in porcine TCs. The significant decrease of both PGRA and PGRB in the corpora lutea during the luteal phase was also observed.

The results of this current study also demonstrated a significant decrease in both investigated progesterone receptors, PGR and PGRMC1, in untreated groups between 48 and 96 h of IVC. Both results may be explained by a lower local availability of progesterone at the luteal phase during porcine ovarian GC differentiation. Taken together, these observations suggest that differential levels of PGR expression may reflect various specificity and/or sensitivity of ovarian cell subpopulations to progesterone administration. On the other hand, a stimulatory effect of E2 on both PGR and PGRMC1 expression was observed. This substantial effect occurs during GC transformation into luteal cells, which may be a result of higher specificity of PGRs to 17beta estradiol and/or the bidirectional effect of this hormone during cell luteinization.

In another study, Kawashima et al. investigated the role of equine chorionic gonadotropin (eCG) and human chorionic gonadotropin (hCG) treatments on the expression of LHCGR and PGR in gilt's GCs and CCs isolated from antral follicles [8]. They found that eCG significantly increased proliferation of porcine GCs and CCs. Moreover, the expression of LHCGR and PGR was accompanied by an increased concentration of progesterone in follicular fluid (FF) prior to LH/hCG secretion. The P4 and PGR play a significant role during FSH-induced LHCGR production in CCs. Our research also demonstrated a stimulatory effect of E2 on PGRs in porcine GCs during short-term culture and cell luteinization. It is known that GC luteinization is significantly associated with the LH/FSH surge and accompanied by P4/E2 action. Taking into account these results, we postulate that eCG, hCG, LH, FSH, P4, or E2 administration is crucial for the proper course of folliculogenesis and maintenance of normal and favorable hormonal profile during porcine COC maturation and/or GC differentiation* in vitro*.

The effect of P4/E2 on COC maturation was well recognized in several species of mammals; however, most recently Toms et al. first demonstrated the role of miRNA in PGR expression during ovarian growth and follicular remodeling. Indeed, it is well known that P4 is an important regulator of target gene transcription during the course of mammalian folliculogenesis [9]. These authors searched for a role of miRNA in GC development during primary culture. They found that treatment of GCs with miR-378-3p significantly decreased* pgr* mRNA as well as the target genes regulated by the progesterone receptor such as* adamts1*,* ctsl1*, and* pparg*. In a similar study, Pan et al. investigated the regulatory effect of miR-378 on* in vitro* CC differentiation, COC maturation and expansion, and aromatase (CYP19A1) expression [10]. They found that CYP19A1 expression was significantly suppressed by miR-378, which was the reason for decreased estradiol synthesis. Moreover, the treatment of CCs with estradiol reversed the inhibitory effect of miR-378 on CC expansion and cell cycle progression. It is suggested that the negative effect of miR-378 may be raised by estradiol. On the other hand, estradiol production is substantially regulated by aromatase, whose activity is suppressed by miR-378. As a result, COC maturation, CC-oocyte “dialog,” and paracrine interaction between these two cells populations appear to belong to one of the important regulatory mechanisms of mammalian folliculogenesis and oogenesis. Taking into account these data, we suggest that P4 and/or E2 may modulate expression of PGRs (PGRA and PGRB) and PGRMC1 as well as the activation of signaling pathways related to PGRs, which is critical for regulation of the proper course of COC maturation, CC-GC growth and differentiation, and follicular remodeling* in vivo* and* in vitro*.

Giguere et al. first identified a subtype of estrogen receptor known as estrogen-related receptor beta [11]. It was shown in several studies that ESRRB plays a significant role in placental development since ESRRB (−/−) deficient animals die in midgestation and display abnormal development, including affected trophoblast proliferation [12]. Recently, Doege et al. explored the role of ESRRB as the pluripotency induced factor responsible for generation of iPSC as well as its influence on stemness marker expression such as SOX2, OCT4, KLF4, c-MYC, PARP1, and TET2 [13]. It was observed that these genes are recruited to the NANOG and ESRRB chromosomal loci. No data exists regarding ESRRB expression in porcine granulosa cells in relation to cell proliferation* in vitro*; however, there are several findings indicating the role of estrogen-related receptors in ovarian cancer growth, progression, and metastasis [14, 15]. Together with our results, we hypothesize that ESRRBs may reflect the function like “growth-differentiation factor,” which regulates both cellular development and transformation. It was found in recent study that ESRRB has no specific ligand, which suggests the potential activation of multiple signal transduction pathways [16]. The results of the current experiments demonstrate substantially increased* esrrb3* mRNA expression at 72 h of IVC, which is related to luteal cell transformation of GCs. Moreover, the stimulatory effect of E2 on ESRRB3 expression after this period suggests that E2 plays a potential role as an ESRRBs agonist in porcine GCs.

In conclusion, E2 upregulates PGR and PGRMC1 expression during both acute and prolonged hormone administration, whereas ESRRB3 mRNA expression is increased after acute and 72 h of prolonged E2 treatment. The response of porcine GCs to E2 administration may be associated with the induction of the luteinization process and cell differentiation* in vitro*.

## Figures and Tables

**Table 1:** Oligonucleotide sequences used for RT-qPCR analysis.

Transcript	Sequence (5′-3′ direction)	Gene accession number	Product size (bp)
PGR	CCATTCGCTTTTCCAGTTAGAC CTGTGAGGATGGAACTTCGT	NM_001166488.1	84 bp
ESRRB3	CCCTGCGGACTATCACTC CCCCTGTCTGTGTCTCTTTG	XM_001928051.6	108 bp
PGRMC1	AGATTCCAGGTCCGTGCC GAACCCCCAAACCCGATAC	NM_213911.1	155 bp
PBGD	GAGAGTGCCCCTATGATGCT ATGATGGCACTGAACTCCT	NM_001097412.1	214 bp
ACTB	CCCTTGCCGCTCCGCCTTC GCAGCAATATCGGTCATCCAT	XM_003124280.3	69 bp
